# HYPOChLorous Acid TEsting Studies in elective groin VASCULAR surgery (HYPOCLATES:VASCULAR): protocol for an ambispective cohort study evaluating a standard change to hypochlorous acid lavage in patients undergoing elective groin vascular surgery

**DOI:** 10.1186/s12893-026-03902-3

**Published:** 2026-06-04

**Authors:** Mika P. Nadvornik, Jonathan Uhl, Tobias Hoch Al Hessen, Niels Siegel, Axel Kramer, Christoph W. Michalski, Dittmar Böckler, Katrin Meisenbacher, Julian C. Harnoss

**Affiliations:** 1 Department of General, Visceral and Transplantation Surgery, Heidelberg University Hospital, Im Neuenheimer Feld 420, Heidelberg, 69120 Germany; 2 Faculty of Medicine, Heidelberg University, Heidelberg, Germany; 3 Department of Vascular and Endovascular Surgery, Heidelberg University Hospital, Heidelberg, Germany; 4 University of Witten/Herdecke, Witten, Germany; 5 Institute for Hygiene and Environmental Medicine, University of Greifswald, Greifswald, Germany

**Keywords:** Hypochlorous acid, Surgical wound infection, Vascular surgical procedures, Intraoperative care, Antisepsis

## Abstract

**Background:**

Surgical site infections remain a frequent and clinically relevant complication following elective open vascular groin surgery, particularly in procedures involving implantation of alloplastic material. Despite routine use in many surgical disciplines, the role of standardized intraoperative wound irrigation in vascular groin surgery is insufficiently defined. Hypochlorous acid is a physiologically occurring antimicrobial agent with broad-spectrum activity and favourable tissue compatibility, making it a promising option for intraoperative wound lavage. However, clinical evidence for its preventive use in elective vascular groin procedures is limited.

**Methods:**

Hypoclates:vascular is a single-centre, ambispective cohort study conducted at Heidelberg University Hospital. Adult patients undergoing elective open vascular groin surgery with arterial exposure are included. A departmental standard change introduced intraoperative wound irrigation with at least 250 ml hypochlorous acid–containing Granudacyn^®^ prior to wound closure, with additional irrigation of alloplastic material where applicable. Patients treated before this change form a retrospective control cohort without antiseptic lavage, while those treated thereafter constitute the prospective cohort. The primary outcome is the incidence of surgical site infections classified according to Centers for Disease Control and Prevention criteria. Secondary outcomes include postoperative morbidity, wound-related complications, antibiotic use, length of hospital stay, and overall morbidity assessed by the Comprehensive Complication Index. Multivariable logistic regression will be used to explore associations between hypochlorous acid-based irrigation and infectious outcomes.

**Discussion:**

This study provides a pragmatic real-world evaluation of standardized hypochlorous acid-based intraoperative wound irrigation in elective open vascular groin surgery. The results will clarify whether this physiologically compatible antiseptic approach is associated with reduced surgical site infections and postoperative morbidity, thereby informing the design of future randomized trials and evidence-based perioperative standards in vascular surgery.

**Trial registration:**

The study was registered on 25 March 2026 in the German Clinical Trials Register (registration identifier: DRKS00039521) prior to data acquisition.

## Background

In vascular surgery, surgical site infections (SSI) represent a severe complication, particularly following procedures involving surgical exposure of the groin [1–4]. The anatomical characteristics of the groin region, including dense lymphatic structures and proximity to bacterial colonisation sites, contribute to an increased susceptibility to postoperative wound complications. In the presence of vascular prostheses or patches, infections are further associated with high revision rates and substantial procedure-related mortality due to foreign material involvement [5–8]. Reported SSI rates following elective open groin vascular procedures vary substantially depending on patient characteristics, urgency of surgery, and degree of tissue ischemia. Contemporary studies report SSI incidences ranging from approximately 5–10% in lower-risk elective procedures to more than 25–30% in higher-risk groin reconstructions involving prosthetic material or advanced peripheral arterial disease [9–11].

While intraoperative wound lavage is commonly performed in many surgical disciplines, inconsistent evidence and guideline recommendations suggest heterogeneous use and lack of uniform standardisation across clinical practice, including vascular surgery and elective groin procedures [12]. Accordingly, available evidence regarding the effectiveness of intraoperative lavage in reducing SSI is limited and inconsistent, and there are no definitive procedure-specific data for elective groin procedures in vascular surgery [13, 14].

The use of antiseptic solutions for intraoperative lavage has been proposed as a strategy to enhance microbial control, yet concerns regarding tissue toxicity and impaired wound healing have restricted their routine use [15, 16]. In this context, hypochlorous acid (HOCl) is of particular interest, as it represents a physiologically occurring antimicrobial molecule generated by the innate immune system and is characterised by broad-spectrum microbicidal activity combined with high tissue compatibility [17, 18].

To date, clinical data on HOCl-based lavage in vascular surgery are scarce. Large-scale observational studies and randomised trials specifically addressing elective groin vascular procedures are lacking [19]. Experimental data demonstrate favourable cytocompatibility [17, 20], supported by animal models and clinical studies in other surgical and wound care settings [21–24]. Importantly, HOCl decomposes into non-toxic by-products, distinguishing it from other antiseptics such as polyhexanide, chlorhexidine (CHX), or octenidine [15].

Although established antiseptics such as CHX and povidone-iodine remain widely used in surgical antisepsis, their intraoperative application as lavage solutions may be limited by concerns regarding local tissue toxicity, fibroblast impairment and delayed wound healing, particularly in vulnerable or ischemic tissue environments [25, 26].

In contrast, the rationale for HOCl-based lavage is not necessarily superior antimicrobial potency in every setting, but rather its potentially favourable balance between antimicrobial activity and tissue compatibility. Experimental and translational studies suggest that HOCl may provide effective microbial control with comparatively low cytotoxicity, favourable wound-healing characteristics and reduced local irritation [25–28].

In addition, HOCl-based irrigation may potentially offer advantages in disrupting microbial biofilms on implanted foreign material such as vascular grafts, patches or clips, which may be particularly relevant in vascular surgery where prosthetic-material associated infections are associated with high morbidity and complex revision procedures [29–33].

Granudacyn^®^, a CE-marked medical device containing HOCl as its active component, is already established in clinical wound management and approved for use in cleansing, irrigation and moistening of acute, chronic, contaminated and surgical wounds, including application in body cavities such as the peritoneal cavity [24, 34]. However, evidence for its preventive application as part of routine intraoperative wound management in elective groin vascular surgery remains limited [12].

The microbial burden required to initiate an SSI is lower than that associated with established wound infection [35]. In elective groin vascular surgery – where prosthetic material is frequently implanted – even modest reductions in bacterial contamination may be clinically relevant. Given its favourable tissue compatibility, HOCl may provide a low-risk option for intraoperative wound lavage as part of routine surgical practice, potentially reducing SSI risk without the adverse effects associated with more aggressive antiseptic agents [18].

## Methods

### Study design

HYPOCLATES:VASCULAR is a single-centre, ambispective, controlled observational cohort study with a retrospective control group conducted at the Department of Vascular and Endovascular Surgery at Heidelberg University Hospital. The study evaluates the clinical impact of a departmental standard change in intraoperative wound management during elective open vascular groin procedures with arterial exposure. Reporting follows the STROBE statement for observational studies [36], and the study protocol was developed in accordance with the SPIRIT guidelines [36, 37].

Patients treated prior to the implementation of the new standard, in whom no antiseptic wound lavage was performed, constitute the retrospective control cohort. The prospective cohort comprises patients undergoing surgery after the standard change who receive intraoperative wound irrigation with a minimum volume of 250 ml of Granudacyn^®^ prior to wound closure. Granudacyn^®^ is a pH-neutral hypotonic electrolyte solution containing stabilised HOCl (50 ppm) and sodium hypochlorite (50 ppm) in equilibrium [38]. In cases requiring implantation of alloplastic material, the graft is additionally irrigated with Granudacyn^®^ before implantation.

The introduction of this standard occurs independently of the study and is not influenced by study participation. No additional diagnostic or therapeutic interventions are required beyond routine perioperative care, including established protocols for antibiotic prophylaxis. Data collection is limited to routinely documented clinical parameters.

### Study population

The study population comprises adult patients (aged ≥ 18 years) undergoing elective open vascular groin surgery with arterial exposure. This cohort represents a clinically relevant population with an increased risk of SSI, particularly in the setting of vascular reconstruction and potential implantation of alloplastic material.

By focusing on a homogeneous group of elective procedures performed in a standardized anatomical region, the study aims to evaluate the effect of HOCl–based intraoperative wound irrigation under real-world clinical conditions while minimizing procedure-related heterogeneity that could confound postoperative infectious outcomes.

Patients unable to receive standard perioperative antibiotic prophylaxis as well as pregnant or breastfeeding individuals were excluded, with pregnancy defined as the period from conception until the end of gestation. Eligibility criteria are summarized in Table 1.

**Table 1:** Inclusion and exclusion criteria

Inclusion criteria	Exclusion criteria
Adults (≥ 18 years old) scheduled for elective open vascular groin surgery with arterial exposure	Contraindication to or inability to receive standard perioperative antibiotic prophylaxis
	Pregnant or breastfeeding women

### Study flow

All patients undergoing elective open vascular groin surgery during the study period will be screened for eligibility and may be excluded according to predefined criteria. Patients treated prior to the implementation of the new intraoperative wound management standard, in whom no antiseptic wound lavage was performed, will constitute the retrospective control cohort.

**Fig. 1:**
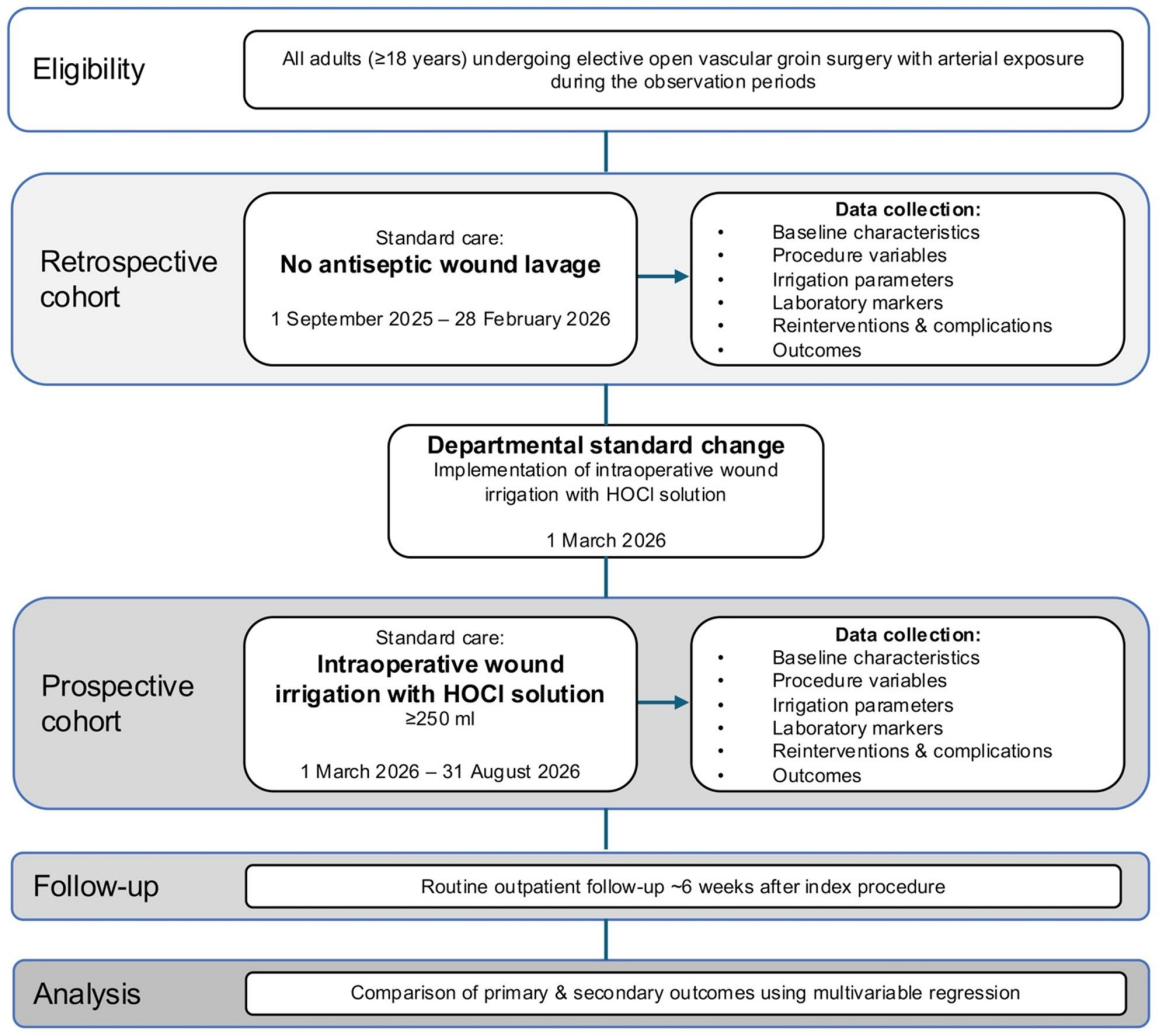
Study flow of HYPOCLATES:VASCULAR

Following the departmental standard change on 1 March 2026, all eligible patients will receive an intraoperative wound irrigation of the groin with HOCl–containing Granudacyn^®^ (≥ 250 ml) prior to wound closure. In cases involving implantation of alloplastic material, the graft will additionally be irrigated with Granudacyn^®^ before implantation. Patients treated after the standard change will form the prospective cohort. The retrospective and prospective observation periods each comprise six months, with the overall study period spanning from 1 September 2025 to 31 August 2026. Post-discharge follow-up is routinely scheduled six weeks after surgery as part of standard vascular surgical aftercare and typically includes clinical wound assessment and routine laboratory testing. Outcomes documented during this routine follow-up, including delayed SSI occurring after hospital discharge, will additionally be captured descriptively where available. A schematic overview of patient inclusion and cohort allocation is shown in Fig. 1.

Study participation requires no additional procedures, diagnostics, or interventions beyond routine clinical care and imposes no additional burden on patients or clinical staff. Consequently, no separate patient information or informed consent is required for the prospective cohort. If the predefined sample size is not reached within the planned observation period, the respective cohort period may be extended.

Apart from the implementation of HOCl-based wound irrigation, perioperative management and SSI prevention standards including antibiotic prophylaxis, skin antisepsis, surgical workflows, and postoperative care remain unchanged throughout both study periods.

### Data collection

Data collection focuses on clinically relevant variables routinely documented as part of perioperative vascular surgical care and considered relevant for postoperative infectious outcomes and risk adjustment. All data will be extracted from the patients’ electronic medical records using the hospital’s digital documentation system (SAP i.s.h.med).

Demographic and baseline clinical variables will include sex, age, body mass index, The American Society of Anaesthesiologists (ASA) physical status classification, smoking status, alcohol misuse, and relevant comorbidities. These comprise diabetes mellitus (requiring treatment with metformin or insulin analogues), chronic kidney disease (requiring dialysis or fluid restriction), chronic obstructive pulmonary disease, immunologic disorders, ongoing immunosuppressive therapy, known bacterial colonization (MRSA, VRE, 3-/4-MRGN), history of previous vascular or groin surgery, and the Charlson Comorbidity Index (Charlson-Index).

Preoperative laboratory parameters will include haemoglobin, white blood cell count, C-reactive protein, serum albumin, and renal function parameters as routinely documented.

Procedure-related variables will include date and type of elective vascular groin surgery with arterial exposure (e.g. endarterectomy, bypass surgery, patch angioplasty, or prosthetic graft implantation), surgical approach, and total duration of the procedure. Severity of peripheral arterial disease and indication for surgery (including Rutherford classification where applicable) will be recorded. Intraoperative data will further include the antiseptic agent used for skin preparation, the degree of wound contamination according to the Centers for Disease Control and Prevention (CDC) classification (clean, clean-contaminated, contaminated, or dirty/infected), perioperative blood loss and transfusion requirements, primary wound closure, placement and type of surgical drains, and implantation of alloplastic material.

Additional intraoperative variables potentially associated with SSI risk will be recorded where routinely documented. These include perioperative glucose management in diabetic patients, perioperative temperature management, use of antimicrobial-coated sutures, preoperative hair removal practice, and other relevant institutional SSI prevention measures.

Details on perioperative antibiotic prophylaxis will be recorded, including the administered agent, timing, redosing, and duration in accordance with institutional standards.

Infection-related outcomes will focus on the occurrence and classification of SSI according to CDC criteria [39], based on clinical and microbiological findings documented during index hospital stay. SSI will be categorized as follows:

1. Superficial incisional SSI: Infection involving only the skin or subcutaneous tissue of the incision, meeting at least one of the CDC-defined criteria.
2. Deep incisional SSI: Infection involving the deep soft tissues (e.g. fascia or muscle layers) of the surgical site, fulfilling CDC criteria.
3. Organ/space SSI: Infection involving any anatomical structure other than the incision that was opened or manipulated during the surgical procedure, fulfilling CDC criteria.

Postoperative complications will include lymphatic fistula, haematoma, seroma, wound dehiscence, postoperative bleeding, graft-related complications, thrombotic or embolic events, myocardial infarction, stroke, pneumonia, pulmonary embolism, urinary tract infection, and need for surgical or radiological reintervention. All complications will be graded according to the Clavien–Dindo classification, and the Comprehensive Complications Index (CCI) will be calculated for each patient.

Postoperative management parameters will include length of intensive care unit stay, need for reoperation or radiological intervention, duration of antibiotic therapy, and total length of hospital stay. Postoperative laboratory monitoring will include routine infection markers as documented during the inpatient period. Follow-up data will be collected for up to six weeks postoperatively as part of routine vascular surgical aftercare.

### Outcomes and hypotheses

Our primary hypothesis states that a standardized intraoperative wound irrigation with HOCl–containing Granudacyn^®^ is associated with a lower incidence of early SSI compared with the previous standard of care without antiseptic lavage.

In accordance, the primary outcome is the incidence of early SSI. The primary endpoint focuses on SSI identified up to hospital discharge, but no later than postoperative day 10 ± 2 days (POD10 ± 2). This restriction was chosen to ensure a robust and uniformly assessable endpoint based on routinely documented inpatient care and to minimize bias related to incomplete capture of post-discharge infections in routine clinical practice.

Importantly, patients undergoing elective vascular groin surgery at our institution routinely receive postoperative follow-up six weeks after surgery as part of standard vascular surgical aftercare, including clinical wound assessment. However, this follow-up does not constitute a study-specific active SSI surveillance programme. Therefore, SSI diagnosed after hospital discharge or beyond POD10 ± 2 during routine follow-up will additionally be captured and reported descriptively in accordance with CDC definitions where documented but will not contribute to the primary endpoint.

The secondary hypothesis is that intraoperative HOCl-based wound irrigation is associated with reduced postoperative morbidity, reflected by shorter hospital stay, reduced antibiotic exposure, and a lower CCI following elective open vascular groin surgery.

Secondary outcomes therefore include postoperative morbidity and clinical course parameters, comprising length of intensive care unit and total hospital stay, duration and type of postoperative antibiotic therapy, wound-related complications (e.g. lymphatic fistula, haematoma, seroma), graft-related or vascular complications, need for surgical or radiological reintervention, and 30-day readmission rates. Overall postoperative morbidity will be quantified using the CCI. All outcomes are detailed in Table 2.

**Table 2:** Major outcomes of HYPOCLATES:VASCULAR

Primary outcome	▪ Surgical site infections (SSI), including superficial incisional, deep incisional, and organ/space infections
Secondary outcomes	▪ Duration of postoperative ICU stay ▪ Total length of hospital stay ▪ Type and duration of postoperative antibiotic therapy ▪ Postoperative day of SSI diagnosis ▪ Reoperations during index hospital stay ▪ Hospital readmissions within 30 days ▪ In-hospital mortality ▪ Overall postoperative morbidity assessed by the CCI ▪ Wound-related complications (e.g. lymphatic fistula, haematoma, seroma) ▪ Postoperative bleeding requiring intervention ▪ Graft-related or vascular complications (e.g. thrombosis, embolism, stenosis) ▪ Need for surgical or radiological reintervention ▪ Nosocomial infections (e.g. urinary tract infection, pneumonia, bloodstream infection) ▪ Myocardial infarction ▪ Pulmonary embolism ▪ Stroke ▪ Bacterial species identified in microbiological samples

### Sample size

Given the exploratory and observational design of this study, no formal confirmatory sample size calculation was performed.

Reported SSI rates after elective open vascular groin procedures range between approximately 5% and 30%, depending on patient risk profile, procedural complexity, and implantation of prosthetic material [9–11]. Based on these rates, an average SSI incidence of approximately 17.5% was assumed for the standard treatment cohort. As data regarding HOCl irrigation in vascular surgery are currently lacking, the anticipated treatment effect was extrapolated from antiseptic irrigation studies in other surgical settings, suggesting a clinically plausible relative SSI reduction of approximately 50%, corresponding to an expected SSI rate of 8.75% in the HOCl cohort [40, 41].

Based on the expected institutional case volume, approximately 160 procedures (80 retrospective and 80 prospective cases) are anticipated during the predefined observation periods. Using a two-sided Fisher’s exact test with an α level of 0.05, this sample size would correspond to an estimated statistical power of approximately 29.9% for detecting the assumed effect size. Accordingly, the present study is not powered for definitive efficacy conclusions but is intended to allow exploratory estimation of potential effect sizes and clinically relevant trends associated with HOCl-based irrigation under real-world conditions, as well as to generate data for the design and formal sample size calculation of future randomized controlled trials.

### Data management

Study data will be recorded using a Microsoft Excel file and stored on an access-restricted folder on a network drive of the Heidelberg University Hospital, which is not accessible from the internet. Data collection will be performed exclusively within the institutional IT infrastructure.

All data will be pseudonymized at the time of extraction from the electronic medical records. The pseudonymization key will be stored separately and accessible only to authorized study personnel. For statistical analysis and dissemination of results, datasets will be fully anonymized to ensure confidentiality and data security.

### Statistical analysis

The unit of analysis in this study is the index surgical procedure. Each elective open vascular groin operation with arterial exposure constitutes one analytical case. In patients undergoing more than one elective open vascular groin procedure during the study period, each procedure is considered a separate case if it represents an independent, planned surgical intervention. Bilateral elective groin operations are analysed as two distinct cases, as each groin represents a separate surgical site with an independent risk profile for surgical site infection and wound-related complications. Reinterventions, revisions, or reoperations performed because of postoperative complications, including surgical site infection, haematoma, lymphatic fistula, bleeding, or graft-related complications, are not considered new cases but are recorded as postoperative outcomes of the corresponding index procedure.

Continuous variables will be summarized as means with standard deviations or as medians with interquartile ranges, depending on their distribution. Group comparisons will be performed using Student’s t-test or appropriate non-parametric tests. Categorical variables will be reported as absolute counts and percentages and compared using the chi-square test or Fisher’s exact test, as appropriate.

The primary analysis will explore the association between intraoperative HOCl–based wound irrigation and the occurrence of SSI using multivariable logistic regression models. A predefined set of clinically relevant covariates will be included in multivariable models to reduce data-driven selection and temporal bias. These covariates include age, sex, Charlson-Index, ASA physical status, wound contamination class, type of vascular procedure, implantation of alloplastic material, diabetes mellitus, smoking status, body mass index, and duration of surgery. Covariate selection is based on established risk factors for SSI in vascular surgery and on variables expected to differ between sequential cohorts.

Effect estimates will be reported as odds ratios with corresponding 95% confidence intervals. All statistical analyses will be exploratory in nature, and p-values will be interpreted descriptively.

Predefined subgroup and sensitivity analyses, for example stratified by procedure type or wound contamination class, will be conducted to assess the robustness of the findings and to explore potential effect modification. Given the exploratory nature of the study and the anticipated number of outcome events, multivariable analyses will be restricted to a limited number of clinically relevant covariates to avoid model overfitting. Variable selection is therefore predefined and hypothesis-driven rather than data-driven.

As the study uses a sequential retrospective and prospective cohort design, temporal confounding cannot be fully excluded. To improve comparability between cohorts, sensitivity analyses using propensity score-based adjustment methods, including propensity score matching or inverse probability weighting, may additionally be performed depending on event numbers and covariate balance.

The extent and pattern of missing data will be reported for all variables. Complete-case analyses will be performed if missingness for key variables is low (< 5%). If missingness exceeds this threshold and is considered plausibly at random, multiple imputation using chained equations may be applied in sensitivity analyses. Variables with extensive missingness or limited clinical relevance may be excluded from multivariable analyses.

## Discussion

### Risk-benefit-assessment

HYPOCLATES:VASCULAR provides a structured clinical evaluation of HOCl–based intraoperative wound irrigation for the prevention of SSI in elective open vascular groin surgery. Even a modest reduction in SSI rates in this high-risk surgical population could yield substantial clinical and health-economic benefits, including fewer wound complications, reduced need for reoperations, shorter hospital stays, and lower overall treatment costs. A reduction in postoperative antibiotic exposure may additionally contribute to antimicrobial stewardship by limiting selective pressure for resistant pathogens.

As a purely observational, non-interventional study, HYPOCLATES:VASCULAR entails no additional diagnostic, therapeutic, or procedural measures beyond established standards of care. The introduction of HOCl-based wound irrigation represents a departmental standard change implemented independently of the study. Potential risks are therefore limited to the known and generally mild adverse effects associated with Granudacyn®.

From an ethical, clinical, and scientific perspective, the conduct of this study is justified. It addresses a relevant and currently unresolved question in vascular surgery – namely, whether physiologically compatible antimicrobial wound irrigation can reduce postoperative infectious morbidity in elective groin procedures. Should HOCl-based irrigation prove beneficial, the findings could support rapid translation into evidence-based perioperative standards without additional burden to patients.

### Study limitations

Several limitations should be considered when interpreting the findings of this study. First, the sequential ambispective cohort design is susceptible to temporal confounding and residual bias despite planned statistical adjustment.

Second, although patients routinely undergo postoperative follow-up approximately six weeks after surgery as part of standard vascular surgical aftercare, the primary endpoint focuses on SSI detected during the index hospital stay and within POD10±2. As routine follow-up does not constitute a study-specific active surveillance programme, delayed postoperative infections occurring after discharge may still be incompletely captured and therefore underrepresented in the primary analysis.

Third, as a single-center observational study, external validity and generalizability may be limited. Accordingly, the findings of this study should be interpreted as exploratory and hypothesis-generating rather than confirmatory evidence of causal efficacy. Nevertheless, the pragmatic real-world design may provide clinically relevant exploratory data to inform future randomized controlled trials.

### Study status

Patient recruitment and data acquisition are ongoing. Clinical outcome analyses will be reported separately following study completion.

## Data Availability

No datasets were generated or analysed during the current study.

## Abbreviations

SSI: Surgical site infection
HOCl: Hypochlorous acid
CHX: Chlorhexidine
ASA: American Society of Anaesthesiologists
MRSA: Methicillin–resistant Staphylococcus aureus
VRE: Voncomycin–resistant Enterococci
3-/4-MRGN: 3–/4–multi–resistant gram–negative bacteria
CDC: Centers for Disease Control and Prevention
CCI: Comprehensive Complication Index
POD: Post–operative day
ICU: Intensive care unit
